# Organizational Climate for Successful Aging

**DOI:** 10.3389/fpsyg.2016.01007

**Published:** 2016-07-04

**Authors:** Hannes Zacher, Jie Yang

**Affiliations:** ^1^School of Management, Queensland University of TechnologyBrisbane, QLD, Australia; ^2^Research Center for Innovation and Strategic Human Resource Management, Jiangxi University of Finance and EconomicsJiangxi, China

**Keywords:** age, attitudes, focus on opportunities, organizational climate, successful aging

## Abstract

Research on successful aging at work has neglected contextual resources such as organizational climate, which refers to employees’ shared perceptions of their work environment. We introduce the construct of organizational climate for successful aging (OCSA) and examine it as a buffer of the negative relationship between employee age and focus on opportunities (i.e., beliefs about future goals and possibilities at work). Moreover, we expected that focus on opportunities, in turn, positively predicts job satisfaction, organizational commitment, and motivation to continue working after official retirement age. Data came from 649 employees working in 120 companies (*M*_age_ = 44 years, *SD* = 13). We controlled for organizational tenure, psychological climate for successful aging (i.e., individuals’ perceptions), and psychological and organizational age discrimination climate. Results of multilevel analyses supported our hypotheses. Overall, our findings suggest that OCSA is an important contextual resource for successful aging at work.

## Introduction

The aging of the workforce and its projected economic and societal consequences have led to an increased interest among organizational researchers and practitioners in the topic of successful aging at work, including ways to maintain and enhance older employees’ motivation, performance, attitudes, and well-being (Finkelstein et al., 2015; Hertel and Zacher, in press). Successful aging at work involves the processes, mechanisms, and conditions that enable employees to achieve favorable subjective and objective work outcomes across the working life span, and particularly at higher ages (Hansson et al., 1997; Kooij, 2015; Zacher, 2015). Zacher (2015) argued that to provide evidence for successful aging at work, researchers need to demonstrate an interaction effect of employee age with personal or contextual resources on work-related outcomes, such that resources explain more variance among older compared to young employees (i.e., a pattern of “differential preservation”; Salthouse, 2006). Extant research on successful aging at work has focused primarily on personal resources (e.g., abilities, motives; Kanfer and Ackerman, 2004) and largely neglected contextual factors that may enhance favorable work outcomes among older employees.

This study, therefore, has three main goals. First, we introduce a new contextual resource for successful aging at work: *organizational climate for successful aging* (OCSA) describes employees’ shared perceptions of the extent to which their organization facilitates successful aging at work. Second, we examine OCSA as a moderator of the relationship between employees’ age and their focus on opportunities. *Focus on opportunities* is a facet of the future time perspective construct that describes individuals’ beliefs about their future goals and possibilities (Cate and John, 2007). Zacher and Frese (2009, 2011) adapted the construct to the work context, argued that it represents a criterion for successful aging at work, and showed that older employees generally perceive fewer opportunities in their remaining time at work than young employees. We expect that OCSA buffers the negative association between employee age and focus on opportunities, even after controlling for individual perceptions of climate and potential alternative explanations (e.g., organizational tenure, age discrimination climate).

Finally, we assume that focus on opportunities, in turn, positively relates to job satisfaction, organizational commitment, and motivation to continue working past official retirement age. *Job satisfaction* has been defined as “a pleasurable or positive emotional state resulting from the appraisal of one’s job or job experiences” (Locke, 1976, p. 1304). Job satisfaction is an important employee attitude because it positively predicts job performance (Riketta, 2008). *Organizational commitment* is defined as employees’ psychological attachment to their organization (Mowday et al., 1979). Consistent with most research on organizational commitment, we focus on employees’ affective (i.e., positive emotional) organizational commitment in this study (Allen and Meyer, 1990). Organizational commitment is positively related to desirable contributions employees make to their work roles, including high job performance and reduced withdrawal behavior (Riketta, 2002). *Motivation to continue working* entails the extent to which employees want to work past their official retirement age (Armstrong-Stassen, 2008; Templer et al., 2010). In the context of an aging workforce, organizations and policy makers are interested in motivating employees to remain employed as long as possible to save costs and enhance productivity (Bal et al., 2012; Earl and Taylor, 2015).

**FIGURE 1:**
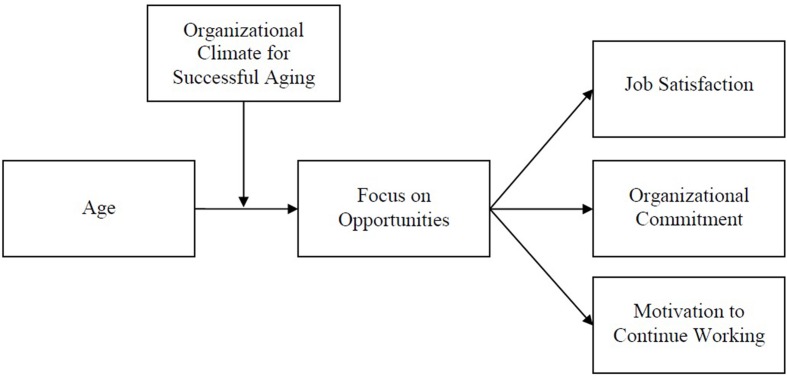
**Conceptual model**.

Overall, our aim with this study is to provide initial evidence for the validity of the OCSA construct and its relations with employee attitudes so that it can be used as a contextual resource in future studies on successful aging at work. Our conceptual model is shown in **Figure 1**.

## Organizational Climate for Successful Aging

A central topic in organizational psychology for more than four decades (Schneider et al., 2013), *organizational climate* is defined as employees’ shared perceptions of their work environment (e.g., policies, norms, procedures, and practices; James and Jones, 1974; Schneider and Reichers, 1983). Organizational climate scores are typically derived by aggregating measures of psychological climate – individual employees’ perceptions of their work environment – across employees within each company (Glick, 1985; James et al., 2008). Researchers have developed several facet-specific organizational climate constructs, including climates for creativity (Ekvall, 1996), safety (Neal et al., 2000), and sustainability (Norton et al., 2014). Research has shown that organizational climates predict relevant employee attitudes, behaviors, and firm outcomes (James et al., 2008; Kuenzi and Schminke, 2009).

Based on the organizational climate literature (Schneider et al., 2013), we define OCSA as employees’ shared perceptions of the extent to which their organization enables successful aging. Aspects of the work environment that facilitate successful aging may include policies and procedures for the equal treatment of employees from different age groups, as well as shared social norms for taking age-related changes in individual characteristics (e.g., increased experience) and personal circumstances (e.g., family and caregiving responsibilities) into account when making work-related decisions (Hansson et al., 1997; Thrasher et al., 2016; Hertel and Zacher, in press).

No empirical research on OCSA exists so far in the organizational and lifespan psychology literatures. However, two recent lines of research have investigated conceptually related phenomena. First, Zacher and Gielnik (2014) showed that top managers’ age interacted with their attitudes toward younger and older employees in predicting employees’ shared perceptions of organizational age culture (i.e., employees’ shared perceptions of the groups of younger and older employees on attributes such as reliable, productive, creative, and flexible). Using data from 66 top managers of small businesses and 274 of their employees, Zacher and Gielnik (2014) showed that the relationship between managers’ age and organizational age culture for older employees was positive among managers with a more positive attitude toward older employees and non-significant among those with a less positive attitude toward older employees. Moreover, the relationship between managers’ age and organizational age culture for younger employees was negative among managers with a less positive attitude toward younger employees and positive among those with a more positive attitude toward younger employees.

A second line of research investigated antecedents and consequences of organizational climates for age discrimination and age diversity. In one study with more than 8,600 employees from 128 companies, researchers showed that age diversity in organizations positively predicted organizational age discrimination climate which, in turn, influenced employees’ collective organizational commitment and overall company performance (Kunze et al., 2011). More recently, the same researchers showed that age-inclusive human resource practices positively predicted organizational age diversity climate in a sample of 93 companies with more than 14,000 employees. Organizational age diversity climate, in turn, positively predicted firm performance and negatively predicted employees’ collective turnover intentions through collective perceptions of social exchange (Böhm et al., 2014).

Organizational climate for successful aging differs from these previously investigated constructs in that it involves employees’ perceptions of their work environment as facilitating the process of successful aging. In contrast, previous studies focused on particular attributes of younger and older employees (Zacher and Gielnik, 2014), or on more specific organizational climate dimensions such as age discrimination and age diversity climates (Kunze et al., 2011; Böhm et al., 2014). Furthermore, the goal of the current study is to examine OCSA as a cross-level moderator of the relationship between employee age and focus on opportunities. In contrast, previous research on age-related climate constructs examined outcomes at the organizational level only and did not examine interaction effects of employee age with organizational climate on employee outcomes (Kunze et al., 2011; Böhm et al., 2014; Zacher and Gielnik, 2014). However, detecting such interaction effects is a necessary prerequisite for demonstrating successful aging (Salthouse, 2006; Zacher, 2015).

## Development of Hypotheses

### Age and Focus on Opportunities

Consistent with the lifespan theory of socioemotional selectivity (Carstensen et al., 1999; Lang and Carstensen, 2002) and previous research on age and occupational future time perspective (e.g., Zacher and Frese, 2009; Zacher et al., 2010; Gielnik et al., 2012, 2016; Bal et al., 2013; Zacher, 2013; Weikamp and Göritz, 2015), we expect that age is negatively related to focus opportunities. Socioemotional selectivity theory suggests that as employees age, their perceptions of remaining time and future opportunities becomes increasingly limited (Carstensen et al., 1999). Older employees are likely to have a lower focus on opportunities than young employees, because both personal and contextual characteristics that may be important for maintaining a focus on opportunities change across the working life span. First, certain relevant personal resources such as physical capabilities (Maertens et al., 2012), motivation to learn (Warr and Birdi, 1998; Kochoian et al., 2016; Kooij and Zacher, 2016), and future time left to pursue new projects (Weikamp and Göritz, 2015) typically dwindle with age, resulting in a lower focus on opportunities among older employees. Second, contextual factors such as age discrimination (Posthuma and Campion, 2009; Griffin et al., 2016), decreased supervisory and organizational support for learning (Mirvis and Hall, 1996), and job design that does not meet age-related changes in resources and needs (Truxillo et al., 2012) may indicate to older employees that their future work-related possibilities are limited.

Hypothesis 1: Age is negatively related to focus on opportunities.

### The Moderating Role of Organizational Climate for Successful Aging

Consistent with the definition and framework of successful aging at work (Zacher, 2015), we further expect that OCSA buffers the generally negative relationship between age and focus on opportunities, such that older employees in organizations with a high OCSA have a higher focus on opportunities than older employees in organizations with a low OCSA; in contrast, we do not expect that OCSA explains much variance in focus on opportunities among young employees.

Issues related to age and aging in the work context are likely to be more salient for older employees and thus there should be a greater need for aging-related contextual resources in this age group (Ward, 1984; Zacher and Frese, 2011). For instance, older employees are more likely to be the targets of negative age stereotypes than younger employees (Chiu et al., 2001; Posthuma and Campion, 2009), and age-related stereotype threat has been shown to have more detrimental consequences for older compared to younger employees’ job attitudes, well-being, and turnover intentions (von Hippel et al., 2012).

Moreover, research based on socioemotional selectivity theory indicates that older compared to younger employees are more emotionally engaged with their organizations because they are more interested in positive and meaningful short-term outcomes than in the achievement of instrumental long-term goals (Ng and Feldman, 2008, 2010). Thus, older employees should benefit more in terms of focus on opportunities from factors at the organizational level. Finally, inter-individual differences in focus on opportunities should become greater with age due to age-related changes in person and contextual resources and demands. This should further increase the likelihood that a contextual resource such as OCSA can explain more variance in focus on opportunities among older compared to young employees.

Hypothesis 2: OCSA moderates the negative relationship between age and focus on opportunities, such that the relationship is weaker when OCSA is high and stronger when OCSA is low.

### Focus on Opportunities and Employee Attitudes

Previous research showed that focus on opportunities is positively associated with employees’ work engagement, job performance, and small business owners’ perceptions of venture growth (Zacher et al., 2010; Gielnik et al., 2012; Schmitt et al., 2013). This research is based on the assumption that a sense of realistic optimism motivates employees to invest in future goals and helps them to achieve positive well-being (Schneider, 2001; Oettingen and Mayer, 2002; Zacher and Frese, 2011). We expect that perceiving many work-related goals and possibilities in the future is also positively associated with job satisfaction, affective organizational commitment, and the motivation to continue working. Employees with a high focus on opportunities should be more satisfied with their job and more committed to their organization, because their work context provides them with meaningful goals and projects to focus on in their future work. Moreover, having a long-term work perspective and meaningful future goals should motivate employees to remain employed beyond official retirement ages.

Hypothesis 3: Focus on opportunities is positively related to (a) job satisfaction, (b) affective organizational commitment, and (c) motivation to continue working past official retirement age.

## Materials and Methods

### Participants and Procedure

Participants in this study were 649 employees from 120 small and medium-sized businesses in Queensland, Australia. Of the participants, 380 (58.6%) were female, and 263 (40.5%) were male (six participants did not indicate their gender). Ages ranged from 18 to 74 years, with an average age of 43.62 years and substantial variation (*SD* = 12.97). In terms of highest level of education, 14 participants (2.2%) did not complete high school, 190 (29.3%) had completed high school, 159 (24.5%) held a technical school degree, 170 (26.2%) held an undergraduate degree, and 111 (17.1%) held a postgraduate university degree (five missing).

This study was carried out in accordance with the recommendations of the University of Queensland’s Behavioural and Social Sciences Ethical Review Committee with written informed consent from all participants. All participants gave written informed consent in accordance with the Declaration of Helsinki. We followed a similar approach to previous empirical studies on organizational culture and climate (e.g., van Dyck et al., 2005; Kunze et al., 2011) and contacted a random sample of 700 small businesses that were listed in a publicly available Australian business database (Dun & Bradstreet’s Company360). A research assistant called each company and asked to speak to a decision maker (e.g., the chief executive officer, manager, or human resource representative) to introduce our study on aging at work. It was not possible to contact 31 companies over the phone because they did not exist anymore or had changed addresses. In 362 cases, it was not possible to talk to a decision maker after three phone calls. Decision makers of 129 companies were not interested in participating, and decision makers in 178 companies indicated their general interest in participating, were sent the study materials (10 employee questionnaires and separate reply paid envelopes), and asked to distribute them to a representative sample of 10 employees.

In total, 661 employees from 120 companies (67.4%) returned between 1 and 10 questionnaires. Consistent with recommendations to use all available data (Newman, 2009), and to detect small and medium-sized effects with adequate statistical power (Scherbaum and Ferreter, 2009), we included all participants who provided complete data on the study variables. This resulted in a final sample of 649 employees from all 120 companies (on average, 5.4 employees per company).

### Measures

Constructs were assessed with relatively short questionnaire scales to minimize time and effort required by participating employees. Employees provided their answers on all scales used in this study on 7-point Likert scales ranging from 1 (*strongly disagree*) to 7 (*strongly agree*).

#### Psychological and Organizational Climates for Successful Aging

Based on the successful aging at work literature (e.g., Hansson et al., 1997; Zacher, 2015), we developed three items to assess climate for successful aging: “Our company is aware of changes that take place with increasing employee age,” “Our company takes age-related changes in employees’ personal circumstances (e.g., family or care responsibilities) into account,” and “Our company is equally supportive of employees from different age groups.” Consistent with recommendations by Chan (1998), we used a referent-shift composition approach, which involves making the organization the referent of the employee level measures (i.e., “In our company, …”). To obtain evidence for the content validity of the scale, we presented 10 subject matter experts (academics with a Ph.D. in organizational or lifespan developmental psychology) with a definition of OCSA and asked them to rate the content measured by each item as “essential,” “useful, but not essential,” or “not necessary” to the performance of the construct (Lawshe, 1975). Lawshe (1975) suggested that an item has acceptable content validity if more than half of the experts indicate that an item is “essential.” Results showed that the percentages of experts who rated the six items to be “essential” were 60, 70, and 70 (30, 20, and 20 percent for “useful”).

An exploratory factor analysis with employees’ ratings on the three items resulted in a one-factor solution that explained 75.40% of the variance, with factor loadings of 0.80, 0.85, and 0.73. We created two variables using the three items. First, we created a psychological climate for successful aging variable for each employee by computing the mean across items. Cronbach’s alpha for the scale was very good (α = 0.84). Second, we computed an OCSA variable by computing a mean score across employees within each company. Aggregation to the company level was justified by intraclass correlation coefficients (ICCs) and *r*_WG(J)_ values that exceeded established cut-off values (Bliese, 2000; LeBreton and Senter, 2008). Specifically, the ICC(1) value was 0.21 (*p* < 0.001), indicating that 21 per cent of the total variance in employee ratings of climate for successful aging can be explained by employees’ membership in their organizations. The ICC(2) value was 0.71, indicating that the reliability of the group means in the sample was satisfactory. Finally, the median *r*_WG(J)_ values (for both uniform and right-skewed distributions) were 0.75, suggesting that the individual-level data can be aggregated to represent OCSA at the company level.

#### Focus on Opportunities

Focus on opportunities was measured with three items from Zacher and Frese (2009), who adapted items from Carstensen and Lang’s (Lang and Carstensen, 2002; Carstensen and Lang, unpublished) future time perspective scale to the work context. The items are “Many opportunities await me in my occupational future,” “I expect that I will set many new goals in my occupational future,” and “My occupational future is filled with possibilities.” Cronbach’s alpha was 0.94. Cate and John (2007) and Zacher and Frese (2009) demonstrated that focus on opportunities was distinct from other future time perspective dimensions, and Zacher et al. (2010) showed that focus on opportunities positively predicted peer ratings of job performance.

#### Job Satisfaction

Job satisfaction was assessed with five items from a reliable and well-validated scale by Judge et al. (1998). Two example items are “I feel fairly well satisfied with my present job” and “I consider my job rather unpleasant” (reverse coded). Cronbach’s alpha for the scale was 0.85.

#### Organizational Commitment

Organizational commitment was assessed with three items from a widely used affective organizational commitment scale developed by Allen and Meyer (1990). Two example items are “I feel emotionally attached to my company” and “I feel a strong sense of belonging to my organization.” Cronbach’s alpha for the scale was 0.93.

#### Motivation to Continue Working Past Official Retirement Age

Motivation to continue working was measured with three items developed by Armstrong-Stassen (2008). Two example items are “If I were completely free to choose, I would prefer to continue working after my official retirement age” and “I expect to continue working as long as possible after my official retirement age.” Cronbach’s alpha for the scale was 0.95.

#### Demographic and Control Variables

We used single items to assess chronological age in years, gender (1 = *male*, 2 = *female*), and highest level of education achieved (1 = *no degree* to 6 = *postgraduate university degree*). We assessed age discrimination climate using a single item based on a four-item scale developed by Robson and Hansson (2007) and adapted by Kunze et al. (2011). As Kunze et al. (2011) demonstrated high homogeneity of the scale items (Cronbach’s α = 0.98, single factor solution), we combined the items of the scale, who all had the same item stem, into a single item: “Age-discriminatory behavior exists in our company (e.g., regarding job assignments, opportunities for development and promotion, performance evaluation, daily leadership).” We controlled for organizational tenure and psychological (i.e., individual perceptions) and organizational (i.e., averaged individual ratings within a company) age discrimination climates to rule out possible alternative explanations for our findings.

### Statistical Analyses

As the data collected in this study had a hierarchical structure (i.e., employees nested within companies), we used multilevel modeling to analyze the data (Hofmann et al., 2000). The organizational level variables (OCSA, organizational age discrimination climate) were centered at the grand (or sample) mean. The employee level predictors (age, psychological climate for successful aging, psychological age discrimination climate) were centered at each company’s (or group) mean (Enders and Tofighi, 2007; Spell et al., 2014). Before the main analyses, we examined the factor structure of all survey items from multi-item scales (psychological climate for successful aging, focus on opportunities, job satisfaction, organizational commitment, and motivation to continue working) by computing multilevel CFAs in MPlus (Muthén and Muthén, 1998–2012). A hierarchical model with the five hypothesized factors at the employee level showed a very good fit to the data (χ^2^[109] = 271.063, *p* < 0.001; CFI = 0.972; TLI = 0.965; RMSEA = 0.047; SRMS_within_ = 0.037). In contrast, a one-factor model did not fit the data well (χ^2^[119] = 3600.959, *p* < 0.001; CFI = 0.395; TLI = 0.308; RMSEA = 0.210; SRMR_within_ = 0.163).

## Results

Descriptive statistics and correlations of the variables at the employee level are shown in **Table 1**. Of note, age was positively related to organizational tenure (*r* = 0.44, *p* < 0.001), job satisfaction (*r* = 0.15, *p* < 0.001), organizational commitment (*r* = 0.13, *p* = 0.001), motivation to continue working (*r* = 0.25, *p* < 0.001), and negatively related to focus on opportunities (*r* = -0.33, *p* < 0.001). Psychological climate for successful aging was positively related to focus on opportunities (*r* = 0.38, *p* < 0.001), job satisfaction (*r* = 0.47, *p* < 0.001), organizational commitment (*r* = 0.49, *p* < 0.001), and motivation to continue working (*r* = 0.13, *p* = 0.001), and negatively related to psychological age discrimination climate (*r* = -0.21, *p* < 0.001).

**Table 1:** Descriptive statistics and correlations.

Variable	*M*	*SD*	1	2	3	4	5	6	7	8
(1) Age	43.62	12.97	–							
(2) Organizational tenure	6.94	7.38	0.44^∗∗^	–						
(3) Psychological climate for successful aging	5.51	1.12	0.01	-0.01	(0.84)					
(4) Psychological age discrimination climate	3.14	1.82	-0.07	0.01	-0.21^∗∗^	–				
(5) Focus on opportunities	4.85	1.43	-0.33^∗∗^	-0.19^∗∗^	0.38^∗∗^	-0.05	(0.94)			
(6) Job satisfaction	5.51	1.02	0.15^∗∗^	0.07	0.47^∗∗^	-0.24^∗∗^	0.33^∗∗^	(0.85)		
(7) Organizational commitment	5.13	1.35	0.13^∗∗^	0.28^∗∗^	0.49^∗∗^	-0.11^∗∗^	0.33^∗∗^	0.54^∗∗^	(0.93)	
(8) Motivation to continue working	4.32	1.82	0.25^∗∗^	0.07	0.13^∗∗^	0.03	0.09^∗^	0.20^∗∗^	0.16^∗∗^	(0.95)

*N = 649. Reliability estimates (α), where available, are shown in parentheses along the diagonal. ^∗^p < 0.05; ^∗∗^p < 0.01*.

**Table 2** shows the results of four multilevel analyses predicting employee attitudes. Overall, the employee and organizational-level predictors explained 20, 28, 23, and 8 percent of the total variance in focus on opportunities, job satisfaction, organizational commitment, and motivation to continue working, respectively. Consistent with the bivariate correlations and in support of Hypothesis 1, age negatively predicted focus on opportunities, after controlling for organizational tenure, as well as psychological climates for successful aging and age discrimination (γ = -0.02, *p* < 0.001). Organizational tenure and psychological age discrimination climate did not significantly predict focus on opportunities, while psychological climate for successful aging positively predicted focus on opportunities (γ = 0.43, *p <* 0.001; see **Table 2**).

**Table 2:** Results of multilevel analyses predicting employee attitudes.

	Focus on opportunities				Job satisfaction				Organizational commitment				Motivation to continue working			
Predictor	*γ*	*SE*	*t*	*p*	*γ*	*SE*	*t*	*p*	*γ*	*SE*	*t*	*p*	*γ*	*SE*	*T*	*p*
Intercept	4.86	0.06	75.31	<0.001	5.52	0.04	139.91	<0.001	5.15	0.06	91.41	<0.001	4.33	0.08	53.17	<0.001
*Employee level predictors*							
Age	-0.03	0.00	-6.86	<0.001	0.02	0.00	4.94	<0.001	0.01	0.00	1.50	0.133	0.05	0.01	6.47	<0.001
Organizational tenure	-0.00	0.01	-0.30	0.764	0.00	0.01	0.75	0.454	0.06	0.01	8.37	<0.001	-0.00	0.01	-0.26	0.792
Psychological climate for successful aging	0.43	0.05	8.48	<0.001	0.32	0.04	8.30	<0.001	0.45	0.05	9.59	<0.001	0.08	0.08	1.10	0.272
Psychological age discrimination climate	0.01	0.03	0.30	0.764	-0.08	0.02	-3.76	<0.001	-0.01	0.03	-0.37	0.710	0.06	0.04	1.56	0.119
Focus on opportunities					0.19	0.03	6.24	<0.001	0.18	0.04	4.73	<0.001	0.20	0.06	3.19	0.002
*Organizational level predictors*							
Organizational climate for successful aging	0.59	0.11	5.48	<0.001	0.41	0.07	6.17	<0.001	0.75	0.09	7.90	<0.001	0.40	0.14	2.91	0.004
Organizational age discrimination climate	-0.00	0.08	-0.01	0.994	-0.08	0.05	-1.70	0.092	0.02	0.07	0.29	0.774	0.09	0.10	0.91	0.366
*Cross-level interactions*							
Age × Organizational climate for successful aging	0.02	0.01	2.02	0.044	-0.01	0.01	-1.13	0.260	0.00	0.01	0.45	0.655	-0.01	0.01	-1.23	0.219
Age × Organizational age discrimination climate	0.00	0.01	0.40	0.692	0.00	0.00	0.40	0.689	0.00	0.01	0.22	0.827	0.02	0.01	1.61	0.108
Null model τ_00_	0.27				0.07				0.03				0.26			
Null model σ^2^	1.76				0.97				1.56				3.06			
Predictor model τ_00_	0.20				0.05				0.16				0.23			
Predictor model σ^2^	1.43				0.70				1.07				2.81			
Model *R*^2^	0.20				0.28				0.23				0.08			

*N = 649 employees nested within 120 companies. Unstandardized coefficients (γ) with standard errors (SE) are shown. τ_00_ = between-person variance; σ^2^ = within-person variance. R^2^ = ([null model τ_00_ + null model σ^2^] – [predictor model τ_00_ + predictor model σ^2^])/(null model τ_00_ + null model σ^2^). Employee level predictors were centered at the group mean, and organizational level predictors were centered at the grand mean*.

**FIGURE 2:**
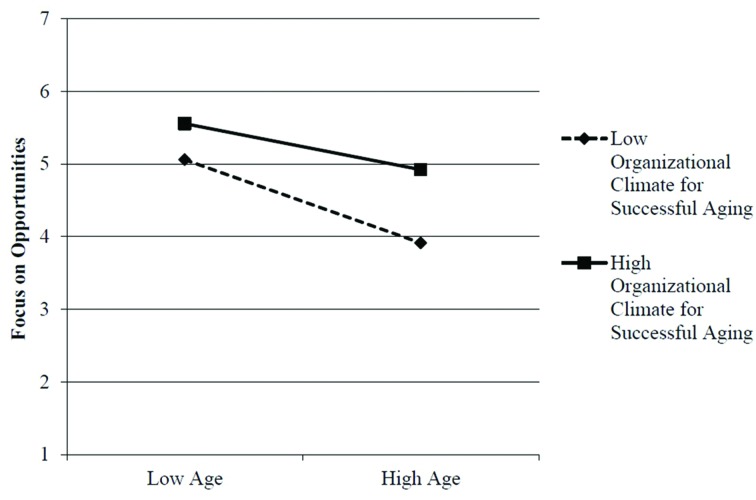
**Organizational climate for successful aging as a moderator of the relationship between employee age and focus on opportunities**.

Hypothesis 2 states that OCSA moderates the negative association between age and focus on opportunities, such that the relationship is weaker when OCSA is high and stronger when OCSA is low. As shown in **Table 2**, both OCSA (γ = 0.59, *p <* 0.001) and the cross-level interaction between age and OCSA (γ = 0.02, *p* = 0.044) significantly predicted focus on opportunities. In contrast, neither organizational age discrimination climate nor the interaction between age and organizational age discrimination climate significantly predicted focus on opportunities. We further probed the significant interaction effect of age and OCSA by regressing focus on opportunities on employee age at more positive (i.e., +1 *SD*) and less positive (i.e., -1 *SD*) values of OCSA. This simple slope analysis indicated that the negative relationship between age and focus on opportunities was weaker in organizations with a more positive OCSA (*B* = -0.02, *SE* = 0.01, *t* = -3.62, *p* < 0.001) than in organizations with a less positive OCSA (*B* = -0.04, *SE* = 0.01, *t* = -6.11, *p* < 0.001). **Figure 2** shows the interaction effect of age and OCSA on focus on opportunities. Consistent with expectations, OCSA explained more variance in focus on opportunities among older compared to young employees. Thus, Hypothesis 2 was supported.

According to Hypotheses 3a–c, focus on opportunities is positively related to job satisfaction, organizational commitment, and motivation to continue working. As can be seen in **Table 2**, focus on opportunities positively predicted job satisfaction (γ = 0.19), organizational commitment (γ = 0.18), and motivation to continue working (γ = 0.20, all *p*s < 0.001), after controlling for age, organizational tenure, as well as psychological climates for successful aging and age discrimination. These findings provide support for Hypotheses 3a–c. We probed the conditional indirect effects of age on job satisfaction, organizational commitment, and motivation to continue working (through focus on opportunities) at high (i.e., +1 *SD*) and low (i.e., -1 *SD*) levels of OCSA using multilevel modeling in MPlus. Results showed that the conditional indirect effects of age on employee attitudes through focus on opportunities were all negative and significant, but, consistent with the moderating effect of OCSA on the age-focus on opportunities relationship, the indirect effects were weaker among employees working in organizations with a high OCSA than the indirect effects among employees in organizations with a low OCSA.

Psychological climate for successful aging positively predicted job satisfaction (γ = 0.32, *p <* 0.001) and organizational commitment (γ = 0.45, *p <* 0.001), but not motivation to continue working (see **Table 2**). While OCSA positively predicted job satisfaction (γ = 0.41, *p <* 0.001), organizational commitment (γ = 0.75, *p <* 0.001), and motivation to continue working (γ = 0.40, *p* = 0.004), the interaction effects of age and OCSA on these outcomes were not significant. Psychological age discrimination climate negatively predicted job satisfaction (γ = -0.08, *p <* 0.001), but not organizational commitment and motivation to continue working. Neither organizational age discrimination climate not its interaction with age significantly predicted these outcomes (see **Table 2**).

## Discussion

### Summary and Interpretation of Findings

In the context of demographic changes, organizational researchers and practitioners are increasingly interested in the factors that help maintain and increase positive outcomes across the working life span, and particularly among older employees. Thus, the first goal of this study was to introduce a new construct and potentially important contextual resource for successful aging in the work context, OCSA. The results of a content validation study suggested that the items of a newly developed OCSA scale were considered essential by a majority of experts in terms of reflecting the construct. Moreover, results of our study showed that the new OCSA scale was homogeneous and reliable, and that the scale scores varied substantially at the individual employee and aggregated organizational levels.

In support of predictive validity, we showed that OCSA was positively associated with focus on opportunities, job satisfaction, organizational commitment, and motivation to continue working past official retirement age, above and beyond psychological climate for successful aging (i.e., individuals’ idiosyncratic perceptions of their work environment) and psychological and organizational age discrimination climates (Kunze et al., 2011). Thus, employees’ shared perceptions of the extent to which their work environment facilitates successful aging are related to important employee attitudes. These main effects of OCSA on relevant outcomes are consistent with organizational climate research in other domains, such as creativity, safety, and sustainability climates (James et al., 2008; Kuenzi and Schminke, 2009; Norton et al., 2014). Interestingly, psychological age discrimination climate was only negatively associated with job satisfaction and, in contrast to previous research by Kunze et al. (2011), organizational age discrimination climate was not significantly associated with aggregated employee attitudes at the organizational level.

The second goal of this study was to examine OCSA as a moderator of the relationship between age and focus on opportunities. We hypothesized that OCSA constitutes a particularly important contextual resource for older compared to young employees, because issues related to age and aging should be more salient and contextual aging resources should be more needed and appreciated by older employees. Consistent with expectations, OCSA buffered the negative relationship between age and focus on opportunities, such that older employees in organizations with a more favorable OCSA reported a higher focus on opportunities than employees in organizations with a less favorable OCSA. In contrast, OCSA explained less variance in young employees’ focus on opportunities. This pattern is consistent with theoretical accounts of successful aging at work (Hansson et al., 1997; Salthouse, 2006; Zacher, 2015).

We found no cross-level moderation effects of OCSA on the relationships between age on the one hand and job satisfaction, organizational commitment, and motivation to continue working on the other. It may be possible that moderation effects of OCSA can only be found for “aging-sensitive” outcomes, that is, variables for which heterogeneity in scores increases as employees get older (Zacher, 2015). For instance, it may be possible that accumulated experiences and age-related contextual factors (e.g., age discrimination) result in greater variation in focus on opportunities scores with increasing age, and that this variation can be explained by personal resources as well as contextual resources such as OCSA. In contrast, young employees are less likely than older employees to vary much in their level of focus on opportunities, and variation in job satisfaction, organizational commitment, and motivation to continue working may also depend less on age and more on work and organizational characteristics that both young and older employees experience.

Finally, in relation to our third goal, we showed that employees’ focus on opportunities positively predicted job satisfaction, organizational commitment, and motivation to continue working, above and beyond the effects of age, organizational tenure, as well as psychological climates for successful aging and age discrimination. These findings extend previous research on the motivational impact of occupational future time perspective (Zacher et al., 2010; Gielnik et al., 2012; Schmitt et al., 2013). Overall, the findings of this study shed new light on contextual resources for successful aging at work and suggest that the new OCSA scale is a valid measurement instrument that could be used or extended in future research in the work and organizational context. Nevertheless, this study has a number of limitations that give rise to several opportunities for future research.

### Limitations and Future Research

A first limitation of the current study is that the design of this study was cross-sectional, and therefore does not allow conclusions about causality or intraindividual age-related changes (i.e., aging) over time. Thus, our study does not allow conclusions about whether OCSA, in combination with employee age, influences employee attitudes or whether employee attitudes, in combination with age, influence OCSA over time. Future research should therefore attempt to collect longitudinal data on OCSA and age- and work-related outcomes. Unfortunately, the barrier to collecting long-term longitudinal data on work and aging is relatively high (Ng and Feldman, 2008). However, Ng and Feldman (2008) suggested that researchers could collect data across critical career transitions. Another possibility may be to collect data in companies undergoing organizational change or to conduct company-level interventions to improve OCSA.

Second, the sample was a convenience sample of small businesses, and only a small selection of employees from each business participated. Even though we asked the company decision makers to distribute the surveys to representative employees within their companies, it may be that these employees had more positive perceptions of their companies than the average employees. Thus, future research should attempt to obtain larger and more representative samples of employees from each company. Alternatively, researchers could focus on managers as informants, which may improve response rates and sample homogeneity. This approach is common in research on organizational culture (van Dyck et al., 2005; Zacher and Gielnik, 2014).

Third, due to space constraints in the questionnaire, and to reduce burden on participating employees, we were not able to control for additional age-related climate constructs that have recently been developed in the literature. Thus, it is not possible to rule out alternative organizational climate explanations of the effects found. For instance, OCSA may overlap with organizational climate for age diversity (Böhm et al., 2014). Future research should examine how these different climate constructs are related and which construct has the strongest effects on successful aging at work. A potentially important construct in this regard is also climate for inclusion, which “involves eliminating relational sources of bias by ensuring that identity group status is unrelated to one’s access to resources, creating expectations and opportunities for heterogeneous individuals to establish personalized cross-cutting ties, and integrating ideas across boundaries in joint problem-solving” (Nishii, 2013, p. 1754). This climate for inclusion may constitute another resource for successful aging especially for older employees who are often confronted with negative age stereotypes held by their coworkers and supervisors (Liden et al., 1996; Posthuma and Campion, 2009; Griffin et al., 2016).

Future research should also control for additional person and contextual factors that were not assessed in the current study. For instance, employees’ work-related attitudes may also depend on the objective and perceived fit between dynamic individual (e.g., abilities) and contextual characteristics (e.g., job demands; Feldman and Vogel, 2009; Zacher et al., 2014). Finally, we did not obtain objective outcomes in this study and instead focused on subjective, self-reported outcomes. While subjective outcomes constitute important criteria for successful aging at work, most developmental researchers agree that the successful aging criteria domain should include both subjective and objective criteria (Heckhausen et al., 2010; Zacher, 2015). Thus, future research could also examine relations of OCSA with occupational health, performance, and the actual decisions to delay retirement.

### Theoretical and Practical Implications

Successful aging involves a complex interplay between age, individual resources (e.g., personality traits, health, education, self-regulatory strategies), and contextual factors (e.g., family, social networks, work; Zacher, 2015; Rudolph, 2016). Researchers interested in successful aging in the work context could develop interactive conceptual models with multiple layers of contextual resources, ranging from specific work characteristics (e.g., task demands, social support) to broader organizational factors such as OCSA and other age-related organizational climates (Farr and Ringseis, 2002). Theory development in this area could adapt a person-environment fit approach to successful aging, which takes dynamic changes in person characteristics as well as contextual factors on multiple levels (e.g., job, team, organization) into account (Feldman and Vogel, 2009; Zacher et al., 2014).

In addition, theory development efforts should focus on gaining a better understanding of successful aging at work as a process that involves interactions between age group differences or intraindividual change over time in employee age and resources. Salthouse (2006) argued that it is necessary that researchers demonstrate a pattern of “differential preservation” across the adult lifespan in order to claim evidence for successful aging. Thus, theories should explain differential implications of age-related resources such as OCSA for both younger and older employees. For instance, an age-related climate construct may exist that has consequences for younger employees only. This could be the case with developmental climate, which involves employees’ shared perceptions of mentoring and coworker support (Spell et al., 2014).

A related implication for future theorizing is to develop a multidimensional model of OCSA, which includes shared perceptions of more specific age-related organizational policies, norms, practices, and procedures related to topic such as recruitment, training, performance appraisal, and promotion. Such a model may include a more abstract higher-order factor similar to the construct examined in the current study, but additionally should include sub-dimensions for different aspects of organizational life that enable or constrain employees’ opportunities for successful aging. Furthermore, such a model should spell out the potential antecedents of OCSA, as well as the mediating mechanisms and boundary conditions of OCSA effects on various employee and company-level outcomes. For instance, leadership behavior shown by supervisors may help translate OCSA into employees’ job satisfaction and organizational commitment. Another possibility is that access to tailored training and development opportunities, or the availability of mentoring roles, mediates the effects of OCSA on older employees’ aging satisfaction and focus on opportunities in the work context. Potential boundary conditions of the effects of OCSA may include company-level factors such as industry, size, and age diversity, as well as employee-level factors such as retirement intentions or promotion and prevention focus.

In terms of practical implications, organizations could attempt to enhance their OCSA as the findings of this suggest that it has beneficial consequences for their employees’ work-related attitudes. For instance, companies could implement human resource strategies with regard to recruitment, training, work design, and promotion that signal to employees that the company is concerned about their successful aging and development at work (Böhm et al., 2013; Böhm and Dwertmann, 2015). Moreover, as organizational culture precedes organizational climate (Schneider et al., 2013), organizational leaders as the main carriers of culture could act as positive role models and help create a work environment that facilitates successful aging (Zacher and Gielnik, 2014). For instance, organizational leaders should prioritize the correct implementation of formal age-related policies (e.g., anti-discrimination policies) into informal practices. Finally, organizations could attempt to raise awareness among their employees for the topic of successful aging, which goes beyond the maintenance and increase of work-related attitudes at higher ages and also includes the use of self-management strategies, promotion of a healthy life style, and retirement planning (Böhm et al., 2013; Hertel and Zacher, in press).

## Conclusion

In summary, we introduced the construct of OCSA in this study and provided preliminary evidence for its validity. Specifically, a content validation study showed that the measure used in this study appears to comprehensively tap relevant aspects of the construct. OCSA further positively predicted a set of work-related employee attitudes, above and beyond the effects of individual employees’ age, tenure, and their idiosyncratic perceptions of their work environment. Finally, and perhaps most interestingly, the study showed that OCSA interacted with employee age in predicting focus on opportunities, such that the negative association between age and focus on opportunities was weakened by high OCSA. Focus on opportunities, in turn, was positively associated with employee attitudes. Overall, these findings suggest that OCSA constitutes an important contextual resource for successful aging in the work and organizational context.

## Author Contributions

HZ designed and carried out the study and wrote the manuscript. JY provided feedback on the study design and the manuscript.

## Conflict of Interest Statement

The authors declare that the research was conducted in the absence of any commercial or financial relationships that could be construed as a potential conflict of interest.
